# Annotations

**Published:** 1908-11-28

**Authors:** 


### ANNOTATIONS.

### Looking Backward.

The occasion of the completion of the new operating theatre at St. Mary's Hospital has been improved in the students' *Gazette* by a symposium of papers contrasting old style with modern in this branch of hospital construction. The old order has yielded place to new, and has left practically no vestige of itself behind, so that the rising generation has no conception at all of how great the changes have been in the last three decades. There is, therefore, a peculiar value in exact descriptions of how surgical operations were conducted in those days. Mr. Edmund Owen contributes a vivid description of the pre-Listerian period, and of the "looseness of hygienic morals" of that time. He is of opinion that the pendulum of surgical practice has swung just a little too far at this time in the other direction, and that there is a suspicion of puritanism in the reaction against the bad old times. Thus he quotes with approval Mr. Stanmore Bishop's dicta on the subject of gloves and masks; similar opinions, it may be remembered, have been advanced in America by Dr. Dukeman. Undoubtedly there is great division of sentiment and practice on this point among the profession in Britain. Even among the teaching staff of the large general hospitals probably a minority as yet wear masks and gloves for all operations. Broadly, it is the seniors who are most conservative in this respect, but whether it is that they are behind the times, or that the juniors are in some cases hyper-fastidious, time alone can decide. The sterilised air, clothes, and so on of certain Continental and American surgeons have not yet gained adherents over here, though sterilised overalls for visitors are now compulsory in many theatres. Still we have moved so far in recent years that he is a bold man who undertakes to assign any limit to the further advances of surgical opinion.

### The Doctor's Motor Car.

In this week's issue of The Hospital will be found the commencement of a series of articles dealing with Motor Cars and Motoring from the point of view of the medical man. We cannot doubt that the establishment of this Section will be regarded with favour by the majority of our readers, since it is evident that almost every medical practitioner of to-day, if he has not already a motor car, is at least a potential motorist. Although there is much difference of opinion as to the right of motor cars to their present somewhat assertive position among road vehicles, and as to the best means of regulating and controlling them, there can be few people nowadays who will deny that the motor car has come to stay, and has indeed become a necessity to many of those who use it. The medical profession, above all others, has benefited to a striking degree by the introduction of motors, and general practice in country districts has been revolutionised by them. Long "rounds," which formerly occupied the better part of the daylight hours, can now be completed before luncheon, and urgent summonses can be obeyed at a moment's notice, to the immense advantage of patient and doctor alike. Surgeons, again, are now by this means independent of Sunday trains and protracted journeys on remote branch lines, and can often complete an urgent operation and return home in the same space of time which was formerly occupied in travelling (with many exasperating changes) no further than the country railway station. Whatever may be the evil effects of the abuse of motor cars by wanton pleasure-seekers, the diffusion of motoring among medical men has been productive of real benefit to the community. The subject of motor cars—their selection, cost, care and maintenance—has thus become a matter of considerable interest and importance to the profession. The termination of the recent successful Motor Show at Olympia, which was attended by many medical practitioners from all parts of the country, seems to be a good opportunity for commencing this new feature of The Hospital; and from now onwards we hope to give our readers regular information on those aspects of motoring which especially concern the medical motorist. Many who already are inclined to suppress their love of horse-flesh on account of the obvious and overwhelming conveniences of motoring, and many more who have a natural mechanical bent, are deterred from buying a car by mere ignorance of the whole subject. It is our intention, through the agency of a practitioner who has identified himself with such matters, to offer advice to intending buyers and weigh the pros and cons of motoring for their benefit; to supply practical hints on the upkeep of cars; and to survey from time to time the entire field of medical automobilism.

### Field Surgery under Marlborough.

Major Howell, whose papers on what may be called the history of the medical service of the British Army are well known, contributes another informative instalment, dealing with the care of the sick and wounded during Marlborough's campaigns, to the current number of the *Journal of the Royal Army Medical Corps*. From it we learn that the "pressed" recruits who took part in that campaign were chiefly vagrants, "men not entitled to vote and members of the criminal classes," and that the only physical standard laid down was that no recruit should be under 5 feet 5 inches in height, and he should be free from rupture. Each cavalry regiment had a surgeon, and each foot regiment a surgeon and a surgeon's mate, the pay being six and four shillings and half-a-crown respectively. The staff surgeons received ten shillings a day. This, by the way, was far better than the pay of their colleagues in the opposing armies, whose unenviable circumstances have been so graphically described by Heister. There was apparently a Director of Hospitals, though Major Howell has not been able to extricate him from the veil of mystery with which the official documents have enshrouded him. It is evident that a carefully thought out organisation for medical relief, for camp hygiene, and for commissariat existed, and that the details "were always under Marlborough's own direct supervision."

